# The wellbeing paradox in Hungarian local sustainable agriculture: a health psychology approach

**DOI:** 10.1186/s12889-022-14643-2

**Published:** 2022-12-12

**Authors:** Ilona Liliána Birtalan, Imre Fertő, Ágnes Neulinger, József Rácz, Attila Oláh

**Affiliations:** 1 Doctoral School of Psychology, ELTE Eötvös Loránd University, Kazinczy utca 23-27, 1075 Budapest, Hungary; 2 Institute of Psychology, ELTE Eötvös Loránd University, Izabella utca 46, 1064 Budapest, Hungary; 3 Institute of Health Promotion and Sport Sciences, ELTE Eötvös Loránd University, Prielle Kornélia u. 47-49, 1117 Budapest, Hungary; 4 Institute of Economics, Centre for Economic and Regional Studies, Eötvös Loránd Research Network, Tóth Kálmán u. 4, 1097 Budapest, Hungary; 5 Hungarian University of Agricultural and Life Sciences, Kaposvár Campus, Guba Sándor utca 40, 7400 Kaposvár, Hungary; 6 Department of Marketing and Tourism, University of Pécs, Rákóczi str. 80, 7622 Pécs, Hungary; 7 Department of Addictology, Faculty of Health Sciences, Semmelweis University, P.O. Box 229, 1444 Budapest, Hungary

**Keywords:** Farmers, Mental health, Work engagement, Work-related stress, Consumer-producer connectivity, Interpretative phenomenological analysis

## Abstract

**Background:**

The literature suggests that farmers’ work involves a number of operational difficulties. Although alternative food networks address the majority of their problems, they can potentially generate new hardships. The aim of this study is to examine the situational and engagement-related work difficulties associated with the everyday world of Community Supported Agriculture (CSA) farmers.

**Methods:**

This study used the health psychology approach, namely interpretive phenomenology, to understand the social determinants of farmers’ working lives in CSA and to explore mental health challenges within the practices of local sustainable farming. To collect data, semi-structured, in-person interviews were conducted with CSA farmers in Hungary.

**Results:**

Our study shows that new modes of consumer-producer connectivity create novel situations and issues which farmers are forced to address. Three personal experiential themes emerge from the data to describe CSA farmers’ work difficulties: (1) Conflicted autonomy; (2) The pressure of boxes; (3) Social overload. The difficulties for CSA farmers seem to be rooted in the economic characteristics of alternative agriculture where farmers organize food production for the satisfaction of consumer needs. In addition, structural conditions require several different CSA farmer roles, which could even be conflicting.

**Conclusion:**

This study provides participants’ perspectives on the health and wellbeing costs of sustainable farming. Newer producer-consumer connections require both time and experience and involve extra effort or skills, but farmers often lack these abilities. The results show how perceptions of work processes relate to the general framework of CSA, which necessitates a distinct strategy for farm management.

**Supplementary Information:**

The online version contains supplementary material available at 10.1186/s12889-022-14643-2.

## Introduction

Interactions among the environmental, social and individual circumstances of farmers have been investigated by a number of academic studies [1–3] and have featured in newspaper articles [4, 5]. Reports and studies from Ghana [6], India [7], Australia [8, 9], France [10], Japan [11] and the U.S. [12, 13] draw attention to the uncertainties of agriculture which are linked to mental health issues. The work of farmers is different from other occupational groups as they have autonomy over their specific work tasks while also being influenced by economic and environmental uncertainty [6, 8, 12]. Such contextual variations can lead to greater stress, increased suicide risk [3, 13]. The relevant literature on the general farming community warns readers that there are concerning levels of physical and mental health problems overall among farmers, which poses a serious risk for the stability of the public food system [14].

To date, numerous studies have described the challenging circumstances farmers work under; namely, the demanding work environment; occupational exposures associated with long working hours as well as the varying weather conditions, climate-change issues [12, 15, 16]. Several pieces of research have emphasized the significance of isolation, stoicism in the face of adversity, family-related conflicts, and the financial hardship farmers may have to deal with [17, 18]. In addition to the mental, emotional, or stress-related problems, farmers’ limited capacity to admit and express mental health difficulties worsens the situation [19]. As farming is a demanding and unpredictable occupation with various risk factors, it can potentially influence individual attributes, perceptions or well-being.

As a response to such dilemmas, some farming has gone through a transition in recent years, with a change in the ownership and management of farms: alternative types of food networks have appeared, offering space for new types of consumer–producer relationships in the food system [20]. This approach to the food system conveys a simple and positive message which provides a pathway for action and advocacy. These alternative food networks (AFN), including community-supported agriculture initiatives (CSAs), farmers’ markets and solidarity purchasing groups, offer alternative food sources for consumers in a local context [21]. Their models, where consumers and farmers are in near proximity to each other, as compared to commodity agriculture, are important as they provide localized opportunities for people to create alternatives to the modern, multinational food system and to exercise their food-related principles [22]. The small-scale farms in AFN, as CSAs, have direct connections between producers and consumers via diversity-based, ecological or alternative food production. That is to say, CSA farmers face a new consumer environment; they take on new work-role demands which need handling, even though there are usually positive features associated with this type of farming at least from the consumer’s point of view [23–25].

CSAs typically produce healthy, organic food and meet the requirements of sustainability by improving the general well-being and overall health of consumers within their own communities. Although a key element for the consolidation of these small-scale food communities is the stable motivation and mental well-being of the producers, AFNs do not automatically provide long term comprehensive, trouble-free solutions to a farmer’s problems [26–28]. Although CSA resulted in a more predictable income flow, it also generated formal and informal duties for the farmer. Our research aimed to explore producer experiences of CSA farming affecting their mental health and work engagement. More specifically, the aim of this study was to understand the working difficulties for farmers which might be encountered in the new modes of consumer-producer connectivity. Our intended contributions to the literature are twofold. First, to better understand the social determinants of farmers’ working lives in new modes of consumer-producer connectivity such as CSA, this study builds on individual stories of farmers and evaluates their realities or ‘lived experiences’ with the help of Interpretative Phenomenological Analysis (IPA). Second, and in relation to the first aim, to identify mental health challenges within the CSA practices of the framework of local sustainable farming.

### Working environment and CSA modes of consumer-producer connectivity

The basis of CSA is that a group of consumers pay in advance to receive a share of healthy, freshly-harvested food every week. Primarily contract-based, CSA usually frames a one-year risk-sharing partnership between farmers and consumers, called members. Food is produced in an agroecological way, and all production and harvesting work is done on the farmer’s land, then put together for the members [24, 29]. To provide a diverse array of local produce, farmers aim to harvest several different types of local produce each week (per box) and, altogether, more than 100 different types of vegetables per year. Vegetables are shared with members each week on particular pick-up days and aim to satisfy the needs of an entire household.

Members range from a dozen people up to a hundred per CSA, and are mostly urban, conscious consumers with a high level of education and in most cases with a family [30, 31]. Moreover, CSA farmers are integrated into the community with the intent of supporting it. Not surprisingly, over the past 30 years, interdisciplinary literature has concentrated on the numerous social benefits of CSA, including its contributions to food security, health, well-being, economic growth, and the transformation of food systems [24, 25, 32, 33].

CSA provides local food, meaning different domains of proximity [34–38]. In this environment, farmers and consumers live relatively close to each other, and all the food is sourced and grown within the region [38, 39]. Moreover, proximity also expresses the direct exchange between producer and consumer, creating direct communication and availability for contact with each other [40]. CSA is based on a direct exchange of plants for money, creating the sense of connectedness and personal belongingness, although high-quality, healthy food is a more important factor for members joining CSA [41].

In addition, proximity is a basis for common values, such as moral economy around the relationships of solidarity between farmers and consumers or ethical concern for the land [42]. Not surprisingly, farmers who are actively involved in a local food environment show commitment to food quality, environmental, and social benefits [15, 33, 43].

Whereas alternative management approaches have significant environmental benefits both on and off farms, working conditions and labour requirements are recognized as suboptimal [44, 45]. Moreover, the increased labour demands of organic agriculture, and the need to negotiate price premiums as well as the relational closeness between consumers and farmers can cause additional stress. Not surprisingly, various research reports call attention to the fact that CSA farmers suffer from their own self- exploitation [46]. These research insights prompt questions about the way new work-related stresses are manifest and approached. Therefore, this study aims to understand the social determinants of farmers’ working lives in CSA and to explore mental health challenges within the practices of local sustainable farming.

## Methods

### Study context

Agriculture plays an important role in Hungary with its share of GDP being the third highest in the European Union. The first three CSA farms were founded around 2010, and fourteen of the existing fifteen CSAs provide fruit and vegetables as their main products [47]. Primarily young, and new farmers take part in CSA initiatives in Hungary. The majority are concentrated around the largest cities. These farmers are primarily skilled organic growers with higher educational backgrounds and these CSAs fed approximately 1,800 people in 2015 based on the first European-wide census on CSA groups [48]. CSAs in Hungary provide predetermined boxes of unprocessed and freshly-harvested products on a weekly basis, mostly satisfying the needs of a household.

Although CSAs have had only rudimentary success in Hungary, the overall data show an approximate 20% increase in turnover for the Hungarian CSA market between 2014 and 2016 and 2015–2017 [49, 50]. While there is no lengthy tradition of CSAs in the country, decisive steps have been taken for their expansion. However, while there are reasons to expect that CSA production and retailing experiences are beneficial for the work situation of individual farmers, there are scant data on the meanings farmers bestow on these CSA experiences.

### Interpretative phenomenological analysis

This study used Interpretative Phenomenological Analysis (IPA) for understanding farmers’ experiences. This is a qualitative methodology originating from, and best known in, health psychology: IPA’s philosophical underpinnings are within phenomenology, hermeneutics and idiography [51, 52]. That is to say, IPA provides a proven, systematic, and phenomenology-based approach to understanding a first-person viewpoint from a third-person perspective.

The limited sample size of most IPA studies allows a micro-level reading of the participant accounts which provides an opportunity for others to gain awareness of these individual experiences. As Miller et al. [53] argue, IPA encourages the development of phenomena and prioritizes diversity linked to lived experience; freedom to explore context; and connection with life narratives. Not surprisingly, IPA requires a combination of empathic engagement and being prepared to probe further into interesting and important aspects of those narratives. In line with it, in IPA investigations, researchers emphasize each participant’s unique idiosyncrasies within shared higher order concepts rather than using the saturation strategy.

Being suitable for the exploration of farmers’ own perceptions, i.e., issues that are continuously relevant, emotionally charged, and a potential cause of dilemma, IPA was appropriate for a deeper understanding of CSA farmers’ work experiences. Personal experiential themes speak to the psychological essence of the whole data set and are illustrated with particular examples taken from individuals [54]. In line with it, the transcript extracts are supplemented with the researchers’ analytic interpretations of the text: giving an account of the data, communicating a sense of what the data are like, and offering an interpretation of the data (to make a case for what they all mean).

### Participants

The study focuses on CSA farmers in Hungary. To collect data, semi-structured, in-person interviews were conducted with six CSA farmers throughout Hungary. To recruit study participants, we used a purposive sampling strategy supplemented by the snowball method based on their relevance to the research questions in order to achieve a detailed, contextual interpretation of people’s personal experiences. Researchers did not determine the sample size a priori, given that sample size is often adaptive and emergent in IPA studies. Two farmers were approached directly – via e-mail or telephone – by the first author after consultation with the Association of Conscious Consumers, which facilitates cooperation and the exchange of best practices between CSA farmers. There were no dropout participants after the authors had obtained the consent.

Participants were required to have been engaged with CSA farming for at least 3 years (the mean was 5.5 years). This enabled us to potentially capture the wider experiences of CSA farmers. The six participants were sufficient to fulfill the idiographic pledge of the IPA and to clarify the common themes among CSA farmers. All the farmers had been involved in both organic farming (and with direct marketing channels, such as farmer’s markets or direct selling) and CSA. Four of them earned their entire household income through CSA, while two of them had other jobs outside CSA (horticulture, direct selling of a special fruit). They had no other jobs outside agriculture. The mean age of participants was 42 years (range from 38 to 47 years), there were three males and three females. All had completed secondary school and two of them had an agriculture-related degree (see Table 1).

**Table 1:** Descriptive data on farmers

	Sex	Age group	Education	Family status	Farming experience	CSA experience (years)	Size of farm	Avg. number of boxes/ week
F1	female	40–45	secondary	married	from childhood	3	5 ha	ca. 70
F2	male	40–45	secondary	married	from childhood	3	5 ha	ca. 70
F3	male	40–45	higher education	relationship	since 2005	7	5.5 ha	ca. 120
F4	female	40–45	secondary	relationship	from childhood	6	2 ha	ca. 40
F5	male	35–40	higher education	married	since 2010	7	2 ha	ca. 120
F6	female	45–50	higher education	single	since 2005	7	1.5 ha	ca. 60

### Procedure

The study design was approved prior to data collection period (2018–2020) by the Institutional Review Board of the University (Ethics protocol approval number: ELTE KEB 2018/202). The first author, as a researcher of this field in Hungary, having earlier focused on CSA consumer experiences, had a prior relationship with some participants and with the Association of Conscious Consumers. These experiences (combined with her health psychologist sensitivity) ensured her ability to closely engage with the research topic.

Farmers were informed of the study aims, that their participation was voluntary, the confidentiality of their information would be ensured, and they could quit at any time without any implications. Interviews were conducted by the first author in the participant’s home so that they might feel comfortable and speak freely. Each interview lasted between 90 and 137 min.

### Measures

The semi-structured interview schedule was designed to elicit farmers’ thoughts, feelings and personal experiences associated with CSA farming. Topic areas were broad so that participants could expand on their responses and include issues they felt to be important. The areas were: becoming a CSA farmer; relating to CSA farming and the community; managing the social environment (CSA family, community, rural community); the meaning of membership; experiences during the course of the CSA season(s) (see the full version of the interview guide in Appendix I.). This interview schedule was used flexibly, with participants largely directing the course of the narrative in order to better explain the issues most salient to them [55].

### Data Analysis

Audio recordings were transcribed and subsequently analyzed using Atlas.ti, a qualitative data analysis software. Transcribed interviews were annotated and analyzed by the first author in line with the principles of IPA [52]. Each transcript was read and re-read: exploratory notes were initially evaluated individually, then across multiple cases. Emergent experiential statements were developed by compiling any exploratory notes that seemed to capture the essence of the experience: this was performed on a case-by-case basis and the initial experiential themes (e.g., agricultural heritage; profession and mind) were arranged and grouped into cohesive, broader superordinate personal experiential themes. Quality control of the data analysis was enhanced through regular supervision by the fourth author. Credibility checks were completed by the second and fourth author which involved cross-checking personal experiential themes. All identifying information was excluded or changed in order to protect participant confidentiality.

## Results

**Fig. 1:**
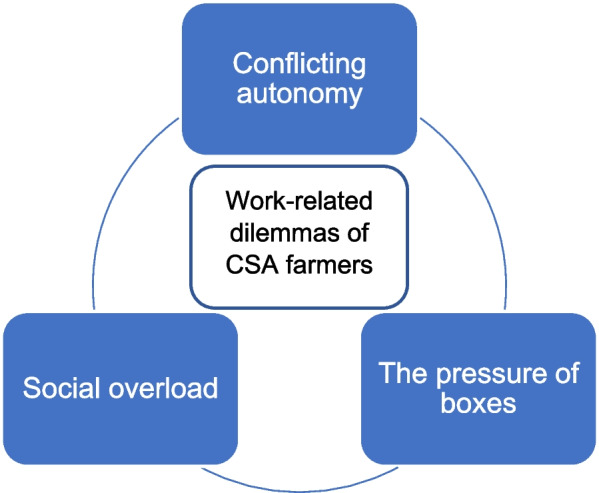
Personal experiential themes for CSA farmers

When CSA farmers were discussing their experiences, the following three personal experiential themes emerged (see summarized in Fig. 1): (1) Conflicting autonomy; (2) The pressure of boxes; (3) Social overload. There was considerable variation in how these were presented, and while there is a certain level of selectivity in choosing illustrative extracts, we sought to present characteristic interview evidence to highlight each theme.

### Conflicting autonomy

Experience of taking part in an alternative food network system had a significant impact on farmers’ work and allowed them to exercise their rights to ethical, balanced, and responsible uses of land and, as well as contributing to a healthy food environment. In practice, farmers’ autonomy takes many forms including agri-environmental schemes, prioritization of non-economic goals in farm strategies, risk-sharing with consumers or not having to commit to a commodity market. This theme of conflicting autonomy emerged across the interviews as participants explained their CSA farm management in relation to the authenticity of their work.

Unfortunately, there is no standard-setting process for farming methods in CSAs. For participants, commitment to excellence encompassed a strong dedication to both one’s work and to high standards (e.g., to grow a variety of crops, increase biodiversity, have a positive impact on the landscape) but it also enhanced their sense of obligation. As an example, Farmer 6 illustrated how CSA operations determined her duties:

This [CSA] is important. I do a good thing, which I like to do, even when I have to break the ice from the water barrel, it’s cold, it’s windy, and I hate it, so despite the physical discomfort, I do because I know how important it is.

Striving to sometimes reach difficult-to-attain outcomes led farmers to become dissatisfied. In fact, participants attempted to set a responsible example. For instance, Farmer 3 experienced disappointments when the harvested vegetables did not meet his own healthy food standards:

It is a professional question - could I manage it …professionally… better? So, these are questions. I do not know, I have demands of myself that I would like to meet. Well, I should probably reconsider my standards, but if I don’t care at all about quality, then why am I doing it?

As farmers became more involved in their own CSA community, possibly taking on leadership roles, they tended to concentrate more on decisions to produce food with the aim of maximizing the size of food boxes for CSA consumers instead of their own rules and norms for good farm management. That is to say, there is a need to monitor the planting calendar to determine what and when to plant, whilst gardening calendars (containing a variety of vegetables) must be followed in order to plan for the harvest. Not surprisingly, Farmer 5 explained how he could not live up to his professional ambitions through CSA as his personal agricultural preferences lay elsewhere:

And even though I also want to improve, the CSA really takes all my energy, and that’s why I am not able to do everything that interests me. Even professionally….

In addition to professional concerns, farmers have to cope with the diversification of vegetables aiming to fulfill the preferences and dietary needs of the members. Yet consumers often cannot appreciate these efforts. On top of that, farmers also experienced the requirement of communicational and educational skills as a tool for influencing members’ attitudes toward a healthy food supply. Farmer 3 illustrated how he attempted to influence his member’s relationship to sweetcorn:

I asked him what he had done, where he had stored it. “Well, I kept it in the pantry” or something similar. I said, Oh My Gosh, this is sweetcorn, it can only be stored for a maximum of one or two days, after which the sugar content degrades. Did you know that? “Well, I had never even heard of that.” was the response.

It appears the CSA environment also creates tension between the financial security of the farmer and customer’s budget. Market logic often constrains the farmer’s room for maneuver causing economic instability of the CSA farm. It serves as a major distress factor for the farmers. As an example, Farmer 3 had to keep opposing financial considerations in her mind:

Honestly, I do try as much as possible financially but vainly. I am stuck in the middle. Members can’t be told to pay fifty thousand HUF [155 US Dollars] a month because they simply can’t pay that much.

Moreover, it seems, it is difficult to balance the competing demands of work and personal projects: changing plans for farming activities or processes to accommodate work demands could challenge work-life boundaries. Farmer 5 categorically pointed out how he delimited his daily farming activities in order to ensure his own free time:

You’re not fair to yourself either, because you shouldn’t work fifteen hours/per day giving you less time for other activities. If you have to grow vegetables every week for thirty-eight weeks, it simply won’t fit in your schedule.

This personal dilemma could be very difficult as farming often requires immediate or sustained action. Sharing risks does not always alleviate the cognitive burden on the individual farmer, but it makes difficult to think about task delegation. Farmer 2 pointed out that he sometimes felt isolated and had to manage a one-man-show:

If you ruin something, you are the only one responsible for it. If you’re not doing something right, for instance watering the radish - if you overwater them, two-thirds of them will crack. That is to say we have to pay so much attention. Nevertheless, we run into totally impossible situations sometimes, which we created for ourselves.

Being their own boss, they aimed to prioritize and represent a better food system, calling for greater clarity and constraints on the use of the land or farming operations. In relation to this, and reflecting the work of Weiler and colleagues, the CSA environment encourages the demonstration of morally-laden behavior (responsibility, fair working environment for employees etc.) during work processes [56]. As an example, Farmer 5 paid attention to those he was actually working with despite the difficulties of finding a likeable workforce in the small-scale agricultural sector:

It’s such a process, that you produce quality organic vegetables for like-minded people who are happy to support this system, and your workers are spending their salary on alcohol? I know it shouldn’t be part of a business approach, but you feel deep down in your bones that you shouldn’t be giving money to people who waste it. It’s not good for a successful business to create this moral approach in you.

### The pressure of boxes

Farmers had to adjust their schedule to the needs of production, to the season and CSA-box numbers, to more intensive work periods because of vegetable quantities or to meet the exact appointments for pick-up days. Additionally, they felt that providing high-quality, compassionate care to consumers was critical, as it is important to match the pre-paid CSA food boxes with members’ expectations. This personal experiential theme is about the pressure of CSA boxes. Farmers described pick-up times as an important dynamic in their farming life: they frequently experienced emotional waves in relation to delivery.

First, precise timekeeping, as a working time characteristic, provides the basis for the CSA farmer-consumer agreement. In addition, as Farmer 6 shared, the non-material part of the agreement also defines the role of a farmer in the CSA model, influencing what level of service they expect to provide:

There was a period when I felt it [CSA] was just a service, because the community did not develop in such a manner. It was better than producing for the farmer’s market, but we only operated as a service provider.

The planning begins with the number of vegetables committed to each box and continues to the finally-harvested vegetables. Observance of what to harvest weekly (in the appropriate box numbers) occasions the need for reliable time measurement. Their weekly harvest duties imply an almost continuously high-level of decision-making, especially in summer. Farmer 2 described how he always has to have a deep understanding of his land and the products he plans to grow:

It’s in the foil tunnel and I think it stays there too. Because on the one hand it grows even in winter, on the other hand, it is always out there, as we also choose varieties that can withstand the cold down to minus twenty degrees [-4^0^ F], and then we bring it into the box from there. Yes, we still produce around five thousand leeks! … And many times, I can’t tell the members why the vegetables are smaller in the box, just talking about the ‘why’, the background behind it… We planted a huge number of vegetables – for example we share three hundred lettuces for a delivery. Now we planted three hundred lettuces, but only about two hundred or two hundred and twenty grew to maturity. We’re really stressed now! I’ve just told X that maybe we should sow ASAP another two or three hundred again because of that. But this is true for anything. You buy and sow the seed, which is not guaranteed to be harvestable, especially not in organic farming.

A CSA’s capacity to produce the appropriate number of vegetables at the right time using environmentally-friendly methods while investing the least amount of time and money is critical to its success. Not surprisingly, Farmer 1 expressed his fears about the variety of foods per box:

You can’t do it… it’s like it’s a huge shame. Let’s say, for example, if there are only three types of veg in the box it is almost as if there is nothing!

In line with the above, meeting consumers often required extra attention, while also triggering revisions in relation to “simplify” farming activities. Furthermore, they had to be aware of the number of empty boxes, returns, bags needed, vehicle condition, as well as the road conditions for transportation. Farmer 4 described how this repetitive pressure begins and influences his day-by-day life:

I’m coming in the evening, so the plan now is that when we get home tomorrow, I’ll water this and that quickly, but then there are seeds to be sown, and then on Monday this and that, and then there is the sowing calendar, right? It restricts me.

Along with the increasing focus on boxes, their experiences were associated with emotions, co-occurrences of both positive (e.g., pride, satisfaction, inspiration, sense of connectedness) and negative effects (e.g., sadness, anger, embarrassment). On the one hand, feedback from CSA members was perceived as useful consumer communication, on the other hand it influenced their emotional life. Farmer 2 stated how personal reactions ensured a direct effect on subjective well-being:

You give them something they have never had before, a new flavour, and you recommend how to prepare this or that vegetable. And then they come back in two weeks to “Wow!” They want another vegetable box because it was so good that the family ate it like it was something incredible!

Moreover, this feedback contributed to their own evaluation of their performance. The members’ responses were bound up with being successful in the way of working the land in a small-scale/organic mode rather than separated from it. Farmer 4 talked about how positive emotions were instrumental for reinforcing his CSA farming activity:

Sometimes I don’t like doing it, but it certainly fills me with a lot of energy. Especially if there is a little positive feedback I receive, then definitely.

Unfortunately, negative emotional responses to farmers were also relevant factors influencing their general mood, determining their psychological state. Farmer 3 described how negative feedback wore him down over time or was, at the very least, a temporary distraction:

If there are only two criticisms in a day then it doesn’t matter that ten people have praised me before them, those two will stay and run around in my head.

### Social overload

Engaging in CSA farming, many farmers experienced a different kind of relatedness including interactions with members, relations with CSA or the rural community, and familial connectedness. Such networks often formed active conduits towards achievement of work satisfaction or amplified dilemmas. Personal, community and even professional relationships were described not only as helping people to feel a sense of belonging or giving meaning, but as reasons for many of the challenges experienced by farmers. This theme emerged as CSA farming impacts on farmers’ social relations.

Farmers wanted to ensure that the benefits of their work accrued to the CSA communities, and they wanted to see the social benefits from cultivating food. The quality of a CSA community is often dictated by the degree of engagement and is affected by community interactions. As Farmer 2 pointed out, the common ground in a CSA is that consumers are like-minded:

I can speak firsthand about mine [members]. I think that anyone who gets into such a community, or wants to get into it, represents a specific perspective.

However, all of the interviewees had certain dilemmas as to what social responsibility they have. As an example, Farmer 6 worried that she was not able to reach a higher level of community engagement via CSA:

It is interesting that the open days often disappoint me … but because of myself, not because of the community. You know, on the open days. I always realise how much more I should be open-minded, or I should be able to control, guide and moderate such get-togethers.

A good community, just as a CSA community, should be cohesive, safe and confident, and farmers should be able to influence the value systems of members around them. Personally knowing members well serves the goal of building social relationships, but it also increases farmers’ awareness of commitments. Farmers explained how they have to manage the mood of their members, occasionally stepping outside their comfort zone. As an example, Farmer 4 reflected on she had to allow a member to leave the CSA (despite the financial loss of losing a member and a fixed one-year contract) so as to protect the social well-being of the community:

She really doesn’t belong to this community; this situation can’t continue like this. If I were her, I would choose that too. And she must be allowed to leave in order to go free with a good feeling on all sides.

Further, participants’ accounts revealed the importance placed on interaction with members. The appropriate psychological distance between farmers and members of their community remains undefined in the CSA framework so that farmers need to set boundaries as to what they are able to manage. But above all, as Farmer 6 commented, it may be that a farmer does not like a member of the community:

There are antipathetic members. It’s hard to say, because they can still be really good member…. Maybe it is not really their fault, maybe they just said something or made a comment that made me feel that way about them, but anyway, what can we do?

Interviewees talked about how they would have preferred to prioritize their own needs, whereas members put their own welfare first, leading to incongruousness. Farmer 1 explained how she grew to hate a customer due to his demands:

He wanted vegetables at unrealistic times that had nothing to do with production and harvesting periods. I was not able explain to him until finally I had to say “NO” to G.! And I have blocked his phone number and NO - there are some people I simply don’t want to have any connection with.

Along with a personal desire to “do better by” their CSA management, participants also expressed certain social dilemmas within the rural community. All of them were committed to CSA membership and the CSA community and were contacted regularly by their urban, conscious consumers. Farmer 4 emphasized the growing social distance in their rural community owing to the CSA social environment:

I love to talk, but I haven got any time for it. I enjoy it maybe too much – so I cannot allow myself to go into the village except one time per week at most. I have no time to chat for hours. But here! This is the CSA community - and how can I express this nicely: there are intelligent people around me here.

Moreover, their production and decision-making processes were influenced by their CSA-related thinking. Such thinking is supported primarily by urban consumers but, in their rural community, perceptions can vary. Not surprisingly, spatial concentration itself creates a favorable environment for CSA [57]. That was emphasized by Farmer 5 who had moved from his home environment into a new area of the country in order to find a more inclusive rural environment:

We saw at a local level … if a young family wants to break out from what’s been going on here for decades, his environment, his family, his neighbours, everyone will pull him down. He will be shouted down… For us, there was no one here next door to say that you are completely out of your mind.

Additionally, all of them were motivated both to become reliable CSA producers and to take care of their families. Unfortunately, there was no model for them to find the work-family life balance between how to engage in and satisfy CSA and family commitments. Continuous availability for consumers along with the specific demands of CSA-related tasks left farmers vulnerable to conflicts between family and work. Farmer 1 illustrated her struggle to switch off from work life:

It is possible that … I will have more foil tunnels… Maybe two people would be enough if I had nothing else to do. … But the fact is that it is still at the expense of the children and at the expense of the family home.

Not surprisingly, family members were a factor that increased their stress. Participation in the family role is made more difficult by virtue of participation in the work role, and the participation in the work role is made more difficult by virtue of participation in the family role. Farmer 5 became a family man, children were born, but he felt that the CSA would not allow him to pay attention to his family:

Because whoever has a family knows how much energy the family needs, and how much attention it needs. At the beginning of a CSA when somebody is doing it alone (as a single man), he has completely different possibilities at the level of daily work; and your personal development is quite different.

## Discussion

Our results harmonize with the literature in many respects. Even organic farmers need to choose between the economic, societal, and ecological aspects of their market [45]. Balancing non-economic and economic benefits for the CSA farmers is a huge challenge, which might influence their sense of personal achievement [33]. While the relationship between consumers and producers is in the very nature of these systems: it is both a requirement and conversely a source of unforeseen challenges for farmers [43, 58]. Moreover, realizing the criteria for economic success, without minimizing social stress and conflicts in relation to farming, could lead to frustration [59].

We are in line with Fraser and colleagues [2], that the stressors of the farming are compounded by the specific framework and economic dynamics of the farm management. The results of this study show how perceptions of work processes relate to the general framework of CSA, which necessitates a distinct strategy for farm management. Doan and colleagues [60] highlighted the importance of investigating the mental health effects of work intensity. In addition, structural conditions require several different CSA farmer roles, which could even be conflicting: agricultural specialist, community organizer, manager and service employee.

We have identified three personal experiential themes: (1) Conflicting autonomy (2) The pressure of boxes; and (3) Social overload in relation to farmer’s experiences from CSA operations affecting their mental health and work engagement. The first personal experiential theme shows how participation contributes to the formation of farmers’ autonomy. On the one hand, farmers in new modes of consumer-producer connectivity can enjoy influencing healthy local food consumption as well as having an impact on the food system as, in effect, being their own manager. On the other hand, operating a CSA farm has a situational influence on how they decide their personal work schedules and procedures limiting the autonomy of their farming operations. Examples of the positive traits accepted by a farming way of life include being near to healthy food, feeling independent in decision-making, and belonging to the respected CSA community of like-minded people; however, sustaining the duties of local sustainable farming intertwined with the everyday obligations to self are obvious sources of stress. The conflicted autonomy theme seems to represent more than the time pressures involved in meeting consumer demands. There is no standard-setting process for farming methods in CSAs and this can cause several work-related stresses in connection with farmers’ work scheduling and decision-making autonomy, also influencing their psychological empowerment [61–63].

Connectedness through ensuring access to food for members is in the very nature of these systems: it is both a requirement and conversely a source of unforeseen challenges for farmers as service providers. Farmers felt that providing a high level of care for consumers is critical. Further, their perceived duties with the diverse weekly harvest might imply the extensive effort to manage the economic dynamics of the CSA in spite of the predictable income flow. This second personal experiential theme indicated that, while farmers were engaged in the weekly box performance, they needed to develop new skills (e.g., communication, education) encapsulating connectedness and efficacy [64]. Moreover, it turned out that very positive feedback or negative responses of consumers on the important pick-up day can lead to significant emotional turmoil [65]. It seems that the meaningfulness of their work combined with their role of social identification unfortunately adds another layer of complexity to their stressors [66].

The last personal experiential theme was that of ‘social overload’. Farming in a CSA has impacts on a farmer’s social connectedness; on their interactions. Farmers’ relationships with consumers require confidence and trust; however, this is based on unequal power and unequal responsibility [67]. Sharing, developing and sustaining relationships with members, or handling them as a community, might suggest a new role requirements – being socially assertive – that is potentially in conflict with being a service employee. This poses a further challenge for the social well-being of farmers. Moreover, connecting to urban members via farmers’ production and decision-making often incurred tensions with those closest in their immediate social environment – defending their uniqueness with CSA, or taking care of their economic and social significance in and with the CSA community. In line with this, interviewees indicated that connectedness conflicts also derived from other relationships such as those with the rural community as well as both work-to-family conflicts and family-to-work conflicts.

New modes of consumer-producer connectivity could strengthen farmers’ sense of mission in various ways, such as grassroot efforts to care about sustainable, healthy, local food and promoting their focus on farming. However, how they experience their work characteristics (as demands or as resources), and what expectations they have as to how they are supposed to behave, provides a basis for further consideration of rural programs in order to maintain health and wellbeing of farmers, which in turn is of paramount importance to the long term food health and food security in the communities [27, 28]. Unfortunately, some of these issues are simply irrelevant within the consumer-farmer relationship and are bound to those areas of production which are hidden from the view of consumers.

### Limitations and future research

Several limitations apply to this study. Firstly, participant recruitment was based upon purposeful and snow-ball sampling which might have introduced a selection bias. Secondly, CSAs working in Central and Eastern Europe were involved, and circumstances could be different in other regions. Future research should investigate in more depth the connections between the CSA framework and farmers’ job characteristics, as these may differ where there are different modes of consumer-producer connectivity. Ecological determinants and their impacts on farmers’ working lives and mental health should also be examined. Another interesting question would be how gender identity shapes values and understanding of self in relation to CSA farming. Probably, the sense of belonging and connectedness to a local community, could improve CSA farmers’ mental health outcomes [64, 68]. This could be compared to conventional farmers. It would also be interesting in future research to compare the time and effort needed to manage a CSA farm with that of a non-organic industrial farm, and to understand the differences between the mindset of farmers at each location.

## Conclusion

New modes of consumer-producer connectivity arrangements are often seen as a better way of producing healthier and more sustainable food supplies [23, 25, 29]. However, while they may actually be better in supporting urban regeneration or providing benefit via increased product margins for famers, they do have consequences for farmers’ subjective well-being and health [15, 69] via development of cooperative and conflicting roles. Most of the findings in farmer mental health related studies are referring to critical factors like: physical problems; remoteness; loneliness and social isolation; rural community attitudes [3, 8, 11, 12]. Our qualitative findings indicate social determinants of mental health are re-established rather than eliminated in the ostensibly transformative CSA context. New modes of consumer-producer connectivity present a new set of work-related discomfort, work-related strain [28, 45, 67]. CSA farmers are constantly caught up, unselfconsciously, in the everyday flow of organic production, community experience, etc. These multiple roles seem to be a barrier in their systematic reflections on the CSA and to be the root of distress.

The difficulties for CSA farmers seem to be rooted in the economic characteristics of alternative agriculture where farmers organize food production for the satisfaction of consumers’ wants, as DeLind [70] warned almost twenty years ago. It seems providing the value-adding basis of farming-centered initiatives vs. conventional food chains does not actually solve the operational problems and the very real pressures of CSA operation in terms of what goods can be produced at a reasonable cost. Farmers’ job satisfaction connects to both the economic challenges and psychological issues, and the solution lies in bringing together knowledge-based considerations with physical capabilities and wider emotional support structures in the CSA model. Moreover in line with Vaderna and colleagues [28] understanding alternative farming subjectivities (and selves) would be particularly beneficial for their long-term existence.

Furthermore, our conclusion also relates to Ge and colleagues [71] findings, namely that high job demands can contribute to lower intrinsic job satisfaction. Accordingly, specific training and development programs could help farmers to improve the skills that would enable them to better demonstrate a sustainable farming role, and work scheduling or decision-making autonomy. It would be recommended on the one hand, to initiate, special educational/skill-development programs to learn about the mental, financial and physical stresses of CSA farming and how to deal with them. On the other hand, a professional exchange program among farmers would be needed to sharpen and add to the skills required, in a CSA. However, it would be important to recognize, that due to the lack of an arbitrator or middleman, the farmer has to deal with the consumer directly. Farmers should learn how to identify patterns of decision-making, or emotional experiences, interpersonal relations, and community settings in relation to CSA farming and, as a result, they could become more self-aware and also realize an improved sense of job involvement.

The positive side of this is that farmers have a chance to inform consumers about the difficulties of farming. The three personal experiential themes identified as inducing stress and requiring coping mechanisms all involve the lack of a buffer between the farmer and the consumer. It might be that in order to make a profit, more consumer commitment is required to ensure a level of income. There are likely to be conflicts between what it costs to produce, what consumers are willing to pay, and these are affected by what is available in the grocery store at a particular price. Maximizing profit would be a matter of optimizing the number of consumers with what products can be affordably grown. Moreover, consumers might be educated about these relationships and the potential supply of products in order to better understand sustainable farming. An educational program could be offered to all those considering joining a CSA.

Methodologically speaking, we argue that first-person perspective qualitative health research [72] should play a significant role within larger contextual debates on health as well as decisions related to farming systems. Better understanding farmers’ perceptions of their everyday work must address the patterns and determinants of their subjective well-being. Using a more interpretive approach aligns with Perceval and colleagues [3] as relevant and contextually-sensitive understanding of farmers’ situations provides a basis for further consideration of agriculture-related health programs and policy supports.

## Supplementary Information

**Additional file 1.**

## Data Availability

The data that support the findings of this study are available from the corresponding author upon reasonable request.
