# Supplementary material for: The wellbeing paradox in Hungarian local sustainable agriculture: a health psychology approach
Source: BMC Public Health. 2022 Dec 12;22:2326. doi: 10.1186/s12889-022-14643-2 (PMC9746150; doi:10.1186/s12889-022-14643-2)
Supplement: Supplementary file 1 — Additional file 1. [file 12889_2022_14643_MOESM1_ESM.pdf]

Appendix I. Full version of the interview guide

AT HOME

- How would you describe your household?

- How specialized is your diet?

- Where else do you like to shop? What foods do you like? How do you cook?

- Tell me about your shopping habits?

EARLIER (before CSA)

- Which of your previous activities can be linked to CSA?

- What's your story, earlier career?

HERE (CSA)

- How did you start with this community?

- Why did you join CSA?

- What attracted you to this opportunity?

HERE (CSA)

- Tell us about your experiences with community farming, be it the very first, later or current one. How have they changed and why?

- What was community farming for you at the beginning of your membership, and what is it now?

- What have you learnt through it, what difficulties have you experienced?

AGRICULTURE

- What does the term ‘community farming difficulties’ (inconveniences) mean to you?

- What is difficult in this field?

- How does production work?

- How do you farm?

- How do you hand over the product?

- What do you pay attention to when handing over?

- What do you pay attention to when planning?

- What do you pay attention to when harvesting?

CHANGE

- What has changed in your life, lifestyle, and views as a result of community farming?

- How has your relationship with farming, the land, crops and members changed?

YOU

- What are you like as a member?

- What are you like as a farmer?

- What helped you to better understand what CSA is all about?

- What does it provide you personally in your life?

- What are your experiences with the CSA community?

- What is a good farmer and why?

OTHERS (inside of the CSA)

- Do you have favorite members?

- Who is the ideal member?

- What is a "normal" member like?

OTHERS (outside of the CSA)

- Who do you meet as new members? Is there anyone who is absolutely amazed by this possibility? How did you inform them and describe the process to them?

- How do people from outside CSA, for whom this is all very "different", see it? Why would it be "different"?

- Who do you accept as members?

- Who should enter into the CSA?

FUTURE

- How do you envisage your future?

- What do you think about the present and future of the CSA?
